# Development of functional beverages from blends of *Hibiscus sabdariffa* extract and selected fruit juices for optimal antioxidant properties

**DOI:** 10.1002/fsn3.331

**Published:** 2016-01-15

**Authors:** Oluwatoyin M. A. Ogundele, Olugbenga O. Awolu, Adebanjo A. Badejo, Ifeanyi D. Nwachukwu, Tayo N. Fagbemi

**Affiliations:** ^1^ Department of Food Science and TechnologyFederal University of TechnologyP.M.B. 704AkureNigeria; ^2^ Department of Human Nutritional Sciences and The Richardson Centre for Functional Foods and NutraceuticalsUniversity of ManitobaWinnipegManitoba R3T 2N2Canada

**Keywords:** Functional beverages, juice formulations, response surface methodology

## Abstract

The demand for functional foods and drinks with health benefit is on the increase. The synergistic effect from mixing two or more of such drinks cannot be overemphasized. This study was carried out to formulate and investigate the effects of blends of two or more of pineapple, orange juices, carrot, and *Hibiscus sabdariffa* extracts (HSE) on the antioxidant properties of the juice formulations in order to obtain a combination with optimal antioxidant properties. Experimental design was carried out using optimal mixture model of response surface methodology which generated twenty experimental runs with antioxidant properties as the responses. The DPPH (1,1‐diphenyl‐2‐picrylhydrazyl) and ABTS [2,2′‐azino‐bis(3‐ethylbenzothiazoline‐6‐sulphonic acid)] radical scavenging abilities, ferric reducing antioxidant potential (FRAP), vitamin C, total phenolics, and total carotenoids contents of the formulations were evaluated as a test of antioxidant property. In all the mixtures, formulations having HSE as part of the mixture showed the highest antioxidant potential. The statistical analyzes, however, showed that the formulations containing pineapple, carrot, orange, and HSE of 40.00, 16.49, 17.20, and 26.30%, respectively, produced optimum antioxidant potential and was shown to be acceptable to a research laboratory guidance panel, thus making them viable ingredients for the production of functional beverages possessing important antioxidant properties with potential health benefits.

## Introduction

Consumers are increasingly better informed about the major role of beverages and foods in diet and health, hence desire functional beverages that contribute to preventing or inhibiting the progression of degenerative diseases caused by oxidative stress (Padayatty et al. 2003; Ozen et al. 2012). Functional beverages are often widely valued (Kausar et al. 2012) with vegetable and fruit beverages also enjoying wide commercial acceptance along with dairy beverages (Davoodi et al. 2013).

Awe et al. (2013) have documented the antioxidant benefits of *Hibiscus sabdariffa* extract ‘HSE’, cocoa and ginger beverage blends as a novel functional beverage. It has also been shown that HSE extracts contain high amounts of protein and other nutrients required for good health (Adanlawo and Ajibade 2006). The aqueous extract of the calyces has been shown to have high acidic and low sugar content (Daramola and Assuni 2006). The sour taste of HSE makes it imperative to use large quantity of sugar and/or artificial sweeteners before consumption (Wong et al. 2002). However, studies have linked high sugar consumption to obesity and type 2 diabetes, and the use of artificial sweeteners such as saccharin, splenda, and aspartame are reported to be carcinogenic (Whitehouse et al. 2008). It thus becomes imperative to find a healthier way of improving the taste of HSE.

Carrot is a rich source of carotenoids which are well known for their antioxidant activity, neuroprotective effect, and ability to improve cognitive development (Ferrari 2004). Orange and pineapple are known for their nutritional value, ascorbic acid content, and rich sensory properties with potential protective action against certain degenerative diseases (Galati et al. 1996; Park et al. 2014).

The mixture design represents an efficient tool to select the best ingredients combination in formulations (Bono et al. 2008). This work describes the formulation and optimization of the four components (pineapple, carrot and orange, and HSE juices) in the development of functional beverages using response surface methodology (RSM), and the determination of the nutritional and antioxidant properties of the optimal beverage.

## Materials and Methods

### Experimental design for the development of beverage formulations

An optimal mixture model design was used with arbitrary lower and upper bounds according to Anderson and Whitcomb (Anderson and Whitcomb 2000). The four independent variables were: pineapple (A), carrot (B), orange (C), and HSE (D). The lower limit (HSE‐10; pineapple‐0; carrot‐0; orange‐0) and upper bound constraints (HSE‐100; pineapple‐40; carrot‐30; orange‐40) for each mixture component were used. The design yielded 15 experimental runs with 5 replicates (Table 1).

**Table 1:** Optimal mixture design matrix for ingredient formulations

Formulation	Pineapple (A)	Carrot (B)	Orange (C)	HSE (D)
F1	15.500	30.000	40.000	14.500
F2	0.000	18.064	24.281	57.705
F3	20.846	30.000	11.406	37.748
F4	21.406	13.398	33.495	31.701
F5	40.000	0.000	0.000	60.000
F6	0.000	30.000	33.300	36.095
F7	0.000	0.000	0.000	100.000
F8	39.816	10.644	13.663	35.877
F9	23.836	17.164	0.000	59.000
F10	24.562	0.000	24.257	51.182
F11	40.000	30.000	12.890	17.110
F12	0.000	30.000	0.000	70.000
aF13	0.000	30.000	0.000	70.000
F14	16.378	0.361	5.604	77.658
F15	40.000	10.000	40.000	10.000
F16	0.000	0.000	40.000	60.000
aF17	0.00	0.00	40.00	60.000
aF18	0.000	0.000	0.000	100.000
aF19	40.000	10.000	40.000	10.000
aF20	40.000	0.000	0.000	60.000

All the mixtures are in percentages.

a Replicates.

### Preparation of pineapple–carrot–orange–HSE beverage formulations

Pineapples, carrots, and oranges were obtained from a local market near the Federal University of Technology, Akure, Nigeria and subjected to commercial maturity index (UNEC 2013). Pineapples (*smooth cayenne*), carrots (*nantes*), and oranges (*sweet oranges*) were sorted, washed, and peeled, and juice extracted from them using Champion juice extractor, model number: KP60PD (Hallelujah Acres, Shelby, North Carolina). The Roselle calyces, obtained from a local market near the Federal University of Technology, Akure Nigeria, were cleaned, crushed, and extracted using calyces: hot water, 50 g: 1000 L for 15 min and filtration of the extract was carried out using a sterilized cheese cloth. Beverage formulations were then prepared according to the experimental design combinations (Table 1). The beverages were filled into sterilized glass bottles and pasteurized at 90°C for 5 min. The samples were cooled and subjected to analysis in the laboratory.

### ABTS scavenging ability, DPPH inhibition, and FRAP antioxidant property

The ABTS [2,2′‐azino‐bis(3‐ethylbenzothiazoline‐6‐sulfonate) radical] scavenging ability of the beverage formulations were determined as described by Re et al. (1999). The trolox equivalent antioxidant capacity (TEAC) was subsequently calculated using trolox as the standard.

$$\%\;Scavenging\;ability\;of\;sample=\left[\frac{Absref-Abssample}{Absref}\right]\times 100$$

The free radical scavenging ability of the beverage samples against DPPH (1,1‐diphenyl‐2‐picrylhydrazyl) free radical was evaluated as described by Gyamfi et al. (1999). The DPPH free radical scavenging ability was subsequently calculated.

$$\%\;Inhibition=\left[\frac{Absorbance\;of\;control-Absorbance\;of\;test\;sample}{Absorbance\;of\;control}\right]\;\times 100$$

The ferric reducing antioxidant property (FRAP) of the beverage formulations was determined by assessing the ability of the extracts to reduce FeCl_3_ solution as described by Oyaizu (1986). The absorbance was measured at 700 nm and FRAP was subsequently calculated using ascorbic acid equivalent.

$$\text{FRAP}=\frac{\left(\mathrm{Absorbance}\;\;\mathrm{of}\;\;\mathrm{Sample}\;*\;\mathrm{Concentration}\;\;,\mathrm{of}\;\;\mathrm{Standard}\;*1000\right)}{(\mathrm{Absorbance}\;\;\mathrm{of}\;\;\mathrm{Standard}\;*\;\mathrm{Concentration}\;\;\mathrm{of}\;\;\mathrm{Sample})}$$

### Total carotenoid, vitamin C, and total phenolic contents

Carotenoid and vitamin C contents were determined according to AOAC (1990) and Awe et al. (2013), respectively. The total phenolic content was determined as described by Singleton et al. (1999).

### Optimum ingredients formulation for the mixed beverage and sensory analysis

The optimization process was carried out using the D‐optimal method as applied by the Design Expert 8.0.3.1 software (Stat‐Ease Inc., Minneapolis, MN, USA). The optimal blend analysis was done using the numerical and graphical methodology. The numerical criteria was set to maximize the values for all the antioxidant properties whereas the graphical optimization was done by superimposing the contour diagrams generated for each of the antioxidant parameter on the same axes; the result was an overlaid contour graph showing the region satisfying the maximum of all the antioxidant properties. The ingredient constraint for the original design was maintained while maximizing the vitamin C, total phenols, DPPH, Carotenoid, and ABTS properties. The predictive regression models developed for each of the criteria were used to develop ternary contour plots to display the effects of the ingredients on the properties. The optimum region was determined by superimposing the contour plots (Palomar et al. 1994) of all the selected criteria for an optimal beverage blend by generating the overlay contour plot for the optimization criteria. From the predicted optimum region obtained, the optimal beverage blend satisfying the optimization criteria was selected. Thus, the chosen blend was reformulated, and varied in treatment. Sodium metabisulphite (100 mg L^‐1^) and sodium benzoate (200 mg L^‐1^) were used as preservatives. During analysis, the samples were refrigerated at 2–4°C for a short period of time.

Sensory evaluation of the beverages was carried out using a 9‐point Hedonic scale (Poste et al. 1991). The sensory panelists consisted of 15 semitrained fruit beverage consumers who evaluated the beverages.

### Statistical analysis

Data were subjected to statistical analysis using response surface methodology (Design Expert version 8.0.3.1 by Stat‐Ease Inc.). Selection of a predictive model to accurately describe each response was based on the quality of fit evaluated by the analysis of variance (ANOVA) statistical package.

## Results and Discussion

### ABTS, DPPH, and FRAP values of the beverage formulations

ANOVA for the special cubic model (Equation 1) of the ABTS antioxidant capacity reveals the model's F‐value as 487.32 and implies that there was a significant effect of the juice blends on the antioxidant property (ABTS) at *P *≤ 0.05, thus showing that the model is an approximate representation of the true system.

(1) $$\begin{array}{cc} \text{ABTS}= & 70.56A+47.71B+155.57C+73.91D-99.64\text{AB}\\ & -294.61\text{AC}-0.30\text{AD}-289.85\text{BC}+33.65\text{BD}\\ & -159.96\text{CD}+331.03\text{ABC}+236.54\text{ABD}\\ & +484.73\text{ACD}+564.64\text{BCD}(1) \end{array}$$

where A = pineapple, B = carrot, C = orange, and D = HSE. *R* ^*2*^ value = 0.9970, adjusted *R* ^*2*^ value = 0.9970.

**Figure 1:**
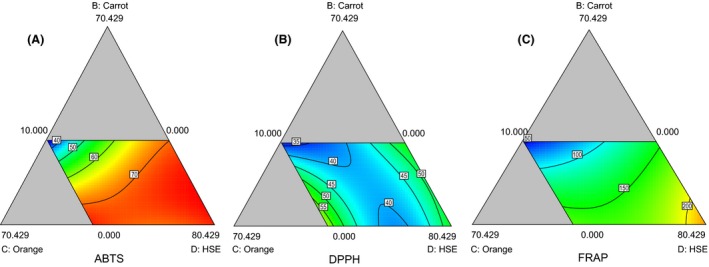
Plots of 2,2′‐azino‐bis(3‐ethylbenzothiazoline‐6‐sulphonic acid)] (A), 1,1‐diphenyl‐2‐picrylhydrazyl (B) and ferric reducing antioxidant potential (C) showing relative interactions among carrot, orange, and HSE beverage blends.

2,2′‐azino‐bis(3‐ethylbenzothiazoline‐6‐sulphonic acid is a chemical compound frequently used by the food industry and agricultural researchers to measure the antioxidant capacities of foods (Huang et al. 2005). The contour diagram indicates that the ABTS scavenging ability was mainly influenced by the HSE content of the formulations. The response plots for ABTS showed that better scavenging potential would be achieved at higher values of the HSE extract. ABTS attained higher values even at low proportions of the carrot, pineapple, and orange juices (Fig. 1A). HSE in combination with *Theobroma cacao* has been reported to contain high levels of antioxidants, which are good for the cardiovascular protection (Awe et al. 2013).

The contour presented in Fig. 1B describes the free radical scavenging ability of the beverage formulations against DPPH and was obtained using a special cubic mixture model which was able to describe 97.87% (*R* ^*2*^ value = 0.9787) of effect of variations in the formulations on DPPH. From ANOVA, the model's F‐value of 21.21 implies that there was a significant effect of the juice blends on the antioxidant property (DPPH) at *P *≤ 0.05. The ability of the formulations to scavenge the radical increased with increasing amounts of HSE.

(2) $$\begin{array}{cc} \text{DPPH}= & 206.90A+140.93B+335.21C\\ & +73.19D-325.10\text{AB}-909.49\text{AC}-283.55\text{AD}\\ & -728.4\text{BC}-130.13\text{BD}-542.09\text{CD}+374.21\text{ABC}\\ & +16.24\;\text{ABD}+908.48\;\text{ACD}\\ & +760.05\text{BCD}(2) \end{array}$$

where A = pineapple, B = carrot, C = orange, and D = HSE. The *R* ^*2*^ value = 0.9787, Adjusted *R* ^*2*^ value = 0.9326.

The actual DPPH scavenging ability of the formulated beverages ranged from 33% to 73%, observed when HSE was at its lower constraint of 10 and highest constraint of 100. This is similar to the scavenging effect of the HSE drink popularly consumed in Egypt, which was determined as 63.9% (Ramadan‐Hassanien 2008). The beverage formulations with higher proportion of HSE were observed to scavenge the DPPH radical faster than those with lower proportion of HSE. High proportion of pineapples contributed more to DPPH values than oranges. Ramadan‐Hassanien (2008) also observed that pineapple extracts had higher antioxidant potential than orange. The contour plot indicates the same trend of increasing scavenging ability with increase in the proportion of HSE.

The ingredient dependent variations in FRAP were best described by the special cubic mixture model presented in Equation 3. The model's F‐value of 7962.23 in Equation 3 implies that there was a significant effect of the juice blends on the antioxidant property (FRAP). The *P* ≤ 0.05 indicates that the model terms are significant and an approximate representation of the true system. The reducing property of the formulations was highest at higher proportions of HSE as observed in Fig. 1C. The reducing power of the beverage formulations were within 61.59 and 247.82 (*μ*mol Fe_2_ SO_4_ g^−1^).

(3) $$\begin{array}{cc} \text{FRAP}= & 86.79A-55.88B+472.\;14C\\ +248.01D+\;27.09\text{AB}-851.07\text{AC}+43.92\text{AD}\\ & -847.74\text{BC}+200.19\;\text{BD}-623.72\text{CD}\\ & +1276.80\text{ABC}-138.52\text{ABD}+713.52\text{ACD}\\ & +1045.74\text{BCD}(3) \end{array}$$

where A = pineapple, B = carrot, C = orange, and D = HSE. *R* ^*2 *^= 0.9999, adjusted *R* ^*2*^ = 0.9998.

It is evident that higher values for FRAP were observed with stronger interaction between the ingredients, as shown by the nonlinear contours. Cissouma et al. (2013) indicated that HSE contain compounds that are capable of donating electrons, which can react with free radicals to convert them to stable products and strongly inhibit radical chain reaction.

### Total phenolic, carotenoid, and vitamin contents of the beverage formulations

**Figure 2:**
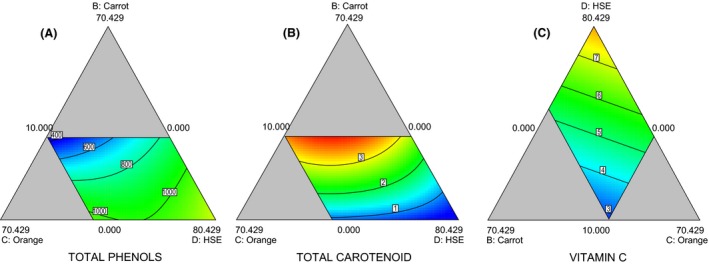
Plots of Total Phenol (A), Total carotenoids (B), and Vitamin C (C) showing relative interactions among carrot, orange, and HSE beverage blends.

Total phenolic content of the varying formulations ranged between 435.58 and 1496 mg GAE 100 g^−1^. ANOVA revealed the model's F‐value as 105.54; implying that the model is significant. *P* ≤ 0.05 indicates that the model terms are significant. The predictive model (special cubic mixture model) for total phenols could explain 99.56% of the influence of variations composition on the total phenolic content of the beverages as presented in Equation 4. The highest phenolic content of 1496 mg GAE 100 g^−1^ was observed when HSE was at its highest, 100%; and lowest value of 435.58 mg GAE 100 g^−1^ when HSE was at 10% constraint (Fig. 2A).

(4) $$\begin{array}{cc} \text{TOTAL} & \;\text{PHENOLIC}\;\text{CONTENT}=\;1379.03A-290.75B\\ & +2929.70C+1488.84D-92.02\text{AB}-6548.18\text{AC}\\ & -1577.16\text{AD}-4004.70\text{BC}+960.65\text{BD}\\ & -4157.40\text{CD}+2228.97\text{ABC}-261.36\text{ABD}\\ +9313.03\text{ACD}+5401.99\text{BCD}(4) \end{array}$$

where A = Pineapple, B = Carrot, C = Orange, and D = HSE; *R* ^*2*^ = 0.9956, adjusted *R* ^*2*^ = 0.9862.

Total phenolic content was observed to be 785 mg GAE 100 g^−1^ with full interaction of all the ingredients. The presence of polyphenolic compounds, such as antroquinones, xanthones, proanthocyanidins, and flavonols, could account for the reasonably strong antioxidant activity in the extracts of stems and leaves (Yen and Chen 1995). Badejo et al. (2014) reported that the scavenging activities of a functional beverage containing germinated and roasted tigernut extracts were significantly enhanced especially with the addition of HSE.

The total carotenoid response to the varying component blends of the beverages was best described as presented in Equation 5. This model could explain 99.99% of the ingredient variations in the beverage formulations. The model's F‐value of 7808.63 implies that the model is significant. A value of *P* ≤ 0.05 indicates that the model terms are significant. The effect of the beverage formulations on the carotenoid content is described by the contour plot in Fig. 2B. The effect of interactions of the design variables indicates highest levels of total carotenoids when carrot extract is included and increases with higher proportions of carrot and shows strong interaction when HSE and carrot are included. The total carotenoids ranged from 0.77 to 3.86 mg g^−1^.

(5) $$\begin{array}{cc} \text{TOTAL} & \;\text{CAROTENOID}\;\text{CONTENT}\;=7.16A+12.74B\\ & +4.18C+0.91D-25.42\text{AB}-24.75\text{AC}\\ & -11.78\text{AD}-14.43\text{BC}-15.02\text{BD}-2.07\text{CD}\\ & +67.80\text{ABC}+53.64\text{ABD}+22.49\text{ACD}(5) \end{array}$$

where A = pineapple, B = carrot, C = orange, and D = HSE. *R* ^*2*^ value = 0.9999, adjusted *R* ^*2*^ = 0.9998.

Research on the antioxidant activity of carotenoids has shown that higher intake of carotenoids leads to a reduced risk of chronic diseases such as cardiovascular diseases and cancer (Rao and Rao 2007). The total carotenoids ranged from 0.77 to 3.86 mg g^−1^ at beverage formulations without carrot and at maximum constraint for carrot (Fig. 2B).

The variation in component combinations and differing influence on the ascorbic content of the mixed beverages had a range of 2.45–8.89 mg g^−1^. The model in equation 6 explains 95.5% of the variations in beverage formulations as it influences vitamin C content. At upper constraint level of HSE, vitamin C was highest at 8.89 mg g^−1^. At 60% HSE, the vitamin C content was 6.89 mg g^−1^ whereas at the lower constraint of HSE (10%), vitamin C dropped to 3.6 mg g^−1^ (Fig. 2C).

(6) VitaminC=4.09A+0.95B+3.57C+8.99D(6)

where A = Pineapple, B = Carrot, C = Orange, and D = HSE; *R* ^*2* ^= 0.9550, adjusted *R* ^*2*^ = 0.9465.

Vitamin C plays an important role as an antioxidant in human health, preventing scurvy and protecting the body against oxidative stress (Padayatty et al. 2003). At low values of carrot, pineapple, and orange juices produced high vitamin C content (Fig. 2C). The linear, or near parallel contours indicated minimal interactions among the ingredients. The contour plots indicate that the vitamin C content depends largely on the quantity of HSE ingredient. The vitamin C obtained for the formulations is comparable to earlier reports of 1.77–4.82 mg g^−1^ in hot and cold HSE beverages (Awe et al. 2013).

### Optimization of ingredient and validation of optimal beverage blend and sensory quality

The comparison between the mean experimental values and predicted antioxidant properties of the chosen optimized beverage formulations showed good agreement (Table 2).

**Table 2:** Predicted and experimental mean values of antioxidant properties for optimal beverage blend

Formulation	Predicted/experimental	Total phenols (mgGAE/100 g)	Vitamin C (mg g^‐1^)	% DPPH
40P‐17C‐17O‐26H	Predicted	655.159	4.307	44.843
	Experimental	512.82 ± 27.14	3.37 ± 0.08	51.34 ± 0.66

P = Pineapple, C = Carrot, O = Orange, H = HSE. 1,1‐diphenyl–2 picrylhydrazyl, DPPH.

The sensory quality of the optimal juice formulations at production is shown in Table 3. The differences between the appearance and aroma of the 100P, 100NP, 70P, and 70NP, and the market sample were not significant. However, the differences in taste and overall acceptability between the optimal juice formulations and the market sample were significant irrespective of presence of preservatives. This confirms the acceptability of the beverages at different concentrations and suggests that the use of preservatives may not influence acceptability by consumers.

**Table 3:** Sensory quality of optimal beverages at production stage

Optimal beverage blend	Appearance	Aroma	Taste	Overall acceptability
100P	7.53 ± 0.98^ab^	7.27 ± 0.81^a^	6.20 ± 0.53^b^	6.40 ± 0.72^b^
100NP	7.33 ± 0.58^b^	7.07 ± 0.31^a^	6.47 ± 0.61^b^	6.40 ± 1.04^b^
70P	7.60 ± 0.20^ab^	7.13 ± 0.13^a^	6.80 ± 0.69^b^	6.80 ± 0.53^b^
70NP	7.50 ± 0.12^ab^	6.73 ± 0.50^a^	6.73 ± 0.61^b^	6.67 ± 0.92^b^
Market sample	8.53 ± 0.31^a^	7.13 ± 0.64^a^	8.60 ± 0.53^a^	8.73 ± 0.31^a^

Values with different superscripts along the same column are significantly different. Each value is the average of three determinations ± SD; 100P = 100% juice with preservatives; 100NP = 100% juice without preservatives; 70P = 70% juice with preservatives; and 70NP = 70% juice without preservatives. The 30% in the 70P and 70NP samples represents the dilution with water.

In conclusion, the best model that describes the ABTS radical, total carotenoids, DPPH inhibiting capacity, ferric reducing antioxidant property, and total phenols, is the special cubic model whereas linear model described the vitamin C contents better. The results indicate that nutrient composition of the optimal beverage blend can be manipulated by changing the ingredient combination. The optimal composition of the beverage formulation was obtained based on each desired antioxidant responses. Beverage blend having formulation of 40% pineapple, 16.5% carrot, 17.2% orange, and 26.3% HSE was found to be optimum. This beverage had total phenols of 512.82 mg GAE 100 g^−1^, Vitamin C content of 3.37 mg g^−1^, and ability to inhibit DPPH as 51.34%. The antioxidant response of the formulations was mainly enhanced by including higher ratios of HSE. The acceptability of the optimized beverage was high, showing that extracts of pineapple, carrots, orange, and HSE can be used as ingredients for the production of a consumer acceptable functional beverage possessing all the important antioxidant properties with potential health benefit.

## Conflict of Interest

The authors declare no conflict of interest.
